# Directly-Observed and Self-Administered Tuberculosis Treatment in a Chronic, Low-Intensity Conflict Setting in India

**DOI:** 10.1371/journal.pone.0092131

**Published:** 2014-03-20

**Authors:** Mrinalini Das, Petros Isaakidis, Edward Armstrong, Nirmala Rani Gundipudi, Ramesh B. Babu, Ihtesham A. Qureshi, Andrea Claes, Anil Kumar Mudimanchi, Nagendra Prasad, Homa Mansoor, Sunita Abraham

**Affiliations:** 1 Operational Research, Médecins Sans Frontières, New Delhi, Delhi, India; 2 District TB Control Office, RNTCP, Khammam district, Andhra Pradesh, India; Institute of Infectious Diseases and Molecular Medicine, South Africa

## Abstract

**Background:**

Limited data are available about tuberculosis treatment models of care for internally displaced populations in chronic, low-intensity conflict zones. This study aimed to detail experiences of a Médecins Sans Frontières tuberculosis programme in Andhra Pradesh-Chhattisgarh border area, India, from January to December 2012.

**Methods:**

The study was a description of two retrospective, observational cohorts receiving category I tuberculosis treatment, either intermittent directly observed treatment (DOT) or daily self-administered therapy (SAT) depending on the security of the area and access to health care services.

**Results:**

A total of 55 and 17 new tuberculosis patients under DOT and SAT respectively, with complete outcomes were included in the study. Most patients registered were new cases suffering from pulmonary, smear-positive tuberculosis. More than half of the patients in both cohorts were cured or completed treatment: 38/55 (69%) patients were successfully treated under DOT compared to 9/17 (53%) under SAT. Of the patients with adverse outcomes, the ratios of loss to follow up: failure: died were 10∶4:3 under DOT and 7∶0:1 under SAT. A much smaller proportion of patients under DOT (18%) were lost to follow up than under SAT (41%).

**Discussion:**

Maximum efforts are required to implement successful tuberculosis control programmes for internally displaced populations in conflict zones. Our study suggests that complete tuberculosis treatment can be given to patients using either intermittent DOT or daily SAT, depending on security and access to health services. National TB programmes should include SAT strategies for tuberculosis treatment as these may be an alternative feasible option in conflict settings.

## Introduction

India contributes to a large share of the global tuberculosis (TB) burden. Of the global annual incidence of 9 million TB cases, 2.3 million cases are estimated to occur in India [1]. The estimated incidence of smear-positive tuberculosis cases in 2012 for Andhra Pradesh (AP) and Chhattisgarh (CG) was approximately 52 and 90 smear-positive cases per 100,000 population respectively. However, limited data are available about TB treatment outcomes within internally displaced populations (IDPs) in chronic, low-intensity conflict zones in the border areas of these two neighbouring states.

Intermittent, directly observed treatment, short-course (DOTS) is administered to tuberculosis patients nationwide, as per the guidelines of Revised National Tuberculosis Control Program (RNTCP) [2]. A DOTS strategy is recommended as the key to successful treatment outcomes for tuberculosis patients [2]. However, there remains a significant proportion of people in India without access to permanent health structures and/or to health care workers that can serve as DOT providers: internally displaced people in conflict zones in border areas and tribal populations are two such groups.

A national plan exists to address treatment of ‘hard-to-reach’ populations and to improve universal coverage of tuberculosis treatment [3], [4]. The need for a patient-centered model of care in order to improve treatment outcomes in tuberculosis patients is also being debated [5]. Multiple studies have been carried out to compare the effectiveness of DOTS and non-DOTS (model of care without direct observation during treatment) treatment delivery for tuberculosis [6]–[8]. Results from these studies emphasized on better treatment outcomes for TB patients, if given complete anti-tuberculosis treatment (ATT) regimen under DOTS-model-of-care. However, a Cochrane review detailed no difference in cure rates between tuberculosis patients taking self-administered therapy (SAT) or receiving DOTS [9]. We were not able to retrieve studies on tuberculosis treatment outcomes among IDPs in chronic conflict zones.

Since 2006, Médecins Sans Frontières (MSF) has been providing primary health care services to the displaced populations living in AP-CG border area. Primary health care services have been offered via mobile clinics in villages and camps for IDPs. Tuberculosis patients are also offered treatment in these areas. The study aims to detail the treatment outcomes in patients receiving either intermittent directly observed treatment (DOT) or daily self-administered therapy (SAT) for tuberculosis, in a conflict setting in India.

## Methods

### Ethics

The study satisfied the criteria for reports using routinely collected programmatic data set by the Médecins Sans Frontières Ethics Review Board (ERB), Geneva, Switzerland. Patient information was anonymised and de-identified prior to analysis. As this was a study of routinely collected monitoring data, informed consent from the patients was not obtained. The named ethics committee specifically approved the study and waived the need for consent.

### Study Design

The study was a description of two retrospective, observational cohorts.

### Setting and Study Population

The programme is located in the border of Chhattisgarh, Andhra Pradesh and Odisha states, an area composed of thickly forested areas, villages and small towns. An ongoing low-intensity chronic conflict between armed extremists and the government has led to displacement of population. The population in these areas suffers from lack of access to primary and secondary health care, including antenatal care and HIV and TB services [10].

The study population consisted of new tuberculosis patients initiated on category I treatment between January and December 2012 and for whom final TB outcomes were available.

The two study areas, Konta and Yampuram, are in two different districts in Chhattisgarh state. There are important differences between the populations that have settled in each of these areas. Konta has a residential semi-urbanized population living in villages. A government Community Health Centre (CHC) in the region provides secondary health care services and tuberculosis treatment. Yampuram is a forested area and has no health services: the nearest accessible public health facility is actually across the border.

### Tuberculosis Treatment Delivery

Due to the difference in access to health services, different treatment delivery strategies were adopted for tuberculosis patients in Konta and Yampuram regions. All the TB patients in Konta received intermittent DOTS as per RNTCP guidelines, under the responsibility of the CHC, supervised by Community Health Workers (CHW). In contrast all the patients in Yampuram were given daily self administered TB treatment (SAT) regimen, as there were no facilities or human resources to observe treatment.

### Treatment Delivery Strategies

#### a. DOT

The patients suffering from active tuberculosis in Konta region received Anti-tubercular treatment (ATT), from a public health structure (Konta CHC). The patients received intermittent ATT which were supervised by a Community Health Worker (CHW) acting as DOTS provider. The CHWs visited the patients’ homes every day to monitor the administration of medicines. Adherence counseling to patients were given by CHWs. Referrals for patients were advised by CHWs, in case of adverse events during treatment. Smear examination and other laboratory investigations were carried out in Konta CHC for these patients, as per the national TB protocol.

#### b. SAT

The tuberculosis patients in Yampuram region received Anti-tubercular treatment (ATT) from the Médecins Sans Frontières (MSF) mobile clinics. Due to the lack of an accessible government health structure nearby, patients could not register or continue the treatment in a designated government health structure. These patients received daily SAT ATT regimen. Once diagnosed with tuberculosis, patients received treatment awareness and adherence counseling by designated staff. The counseling was similar to the DOT patients, however the importance of adherence to treatment was emphasized for patients on SAT. The patients then were given medicines for 2 months (6 weeks of medication and 2 weeks of a buffer supply) and advised to return to a fixed place where the mobile clinic would visit after 6 weeks. During each follow-up assessment, smear and other laboratory examinations were carried out, and results were given to the patient. Patients were asked to report every 6 weeks. However, they were encouraged to visit the mobile clinic every week, in case of illness/need. Lost to follow up tracing and additional supportive counseling were provided to patients interrupting treatment or temporarily lost to follow up depending on the security situation.

### Treatment Regimens

#### Patients under DOT

Patients in Konta region received tuberculosis treatment from Konta CHC, based on national tuberculosis guidelines (RNTCP) [3]. The medicines were given in intermittent doses supervised by Community Health Workers as DOT providers for the patients.

#### Patients under SAT

Patients in Yampuram region received tuberculosis treatment from Médecins Sans Frontières (MSF), based on MSF guidelines [19]. The medicines were given in daily doses with patients, who were empowered to be responsible for their tuberculosis treatment completion.

Box 1 and 2 provide detailed information on treatment delivery strategies and, regimens respectively.

### Data Collection and Analysis

Demographic and clinical information were systematically recorded in patient files. The clinical data being routinely collected for each patient, including treatment and laboratory data, were entered into an electronic database. Data from all TB patients initiated on treatment between January 2012 and December 2012 with final treatment outcomes were included in the analysis. Simple descriptive statistics, univariate and bivariate analysis and Kaplan Meier survival curves were used. Successful outcomes were defined as bacteriologically confirmed cure or treatment completion and adverse outcomes defined as death, failure or loss to follow up.

## Results

### Patient Characteristics

A total of 89 patients were started on treatment during the study period. Records of the HIV status were unavailable for the great majority of the patients (86%) in the study. Of 10 TB patients with known HIV status, seven were co-infected HIV. Seventeen patients were on category II treatment and were excluded from the study. Fifty-five and 17 new tuberculosis patients under DOT and SAT respectively were on category I treatment and were included in the study (Table 1). The mean age of TB patients was 38 years under DOT and 32 years under SAT. Males were in majority in both the regions: 35/55 (64%) in DOT and 10/17 (59%) in SAT areas. The ratios of pulmonary smear-positive: pulmonary smear-negative: extra-pulmonary TB were 41∶9:5 in the DOT group and 8∶4:5 in the SAT group.

**Table 1:** Demographic and treatment outcome details of patients receiving DOT or SAT for tuberculosis in Andhra Pradesh/Chhattisgarh, India.

Explanatory Variable	Patients receiving DOT(N = 55) n (%)	Patients receiving SAT(N = 17) n (%)	T-test value/Chi-square test value(p-value)
**Age** (years, mean ± SD)	38.85±13.01	32.71±16.01	1.61 (0.11)
**Sex of patients**			
Male	35 (63.6)	10 (58.8)	0.13 (0.72)
Female	20 (36.4)	7 (41.2)	
**Category of TB**			
Pulmonary, smear-positive	41 (74.5)	8 (47.1)	–
Pulmonary, smear-negative	9 (16.4)	4 (23.5)	
Extra-pulmonary	5 (9.1)	5 (29.4)	
**Treatment outcome**			
Cured	29 (52.7)	4 (23.5)	–
Completed	9 (16.4)	5 (29.4)	
Loss to follow up	10 (18.2)	7 (41.2)	
Failure	4 (7.3)	–	
Died	3 (5.5)	1 (5.9)	
**Final outcome**			
Successful	38 (69.1)	9 (52.9)	1.49 (0.22)
Adverse	17 (30.9)	8 (47.1)	
**Treatment interruption** †			
No interruption/less than 15 days	28 (73.7)	5 (55.6)	–
15 days and above	10 (26.3)	4 (44.4)	
**Duration of treatment** †			
Category 1(weeks, mean ± SD)	26.47±3.30	32.67±8.35	3.7 (**0.01**)

† Patients with successful treatment outcomes (cured and completed).

### Treatment Outcomes

More than half of the TB patients in both cohorts were either cured or had completed their treatment: 38/55 (69%) patients were successfully treated under intermittent DOT region compared to 9/17 (53%) under SAT. Of the patients with adverse outcomes, the ratios of loss to follow up: failure: died were 10∶4:3 under DOT and 7∶0:1 under SAT. A much smaller proportion of patients under DOT (18%) were lost to follow up than under SAT (41%).

The mean treatment duration of treatment was shorter for patients under DOT than SAT: 26.5 weeks under DOT vs. 32.7 weeks under SAT. Among the patients with successful treatment outcomes, 10/38 (26%) patients under DOT and 4/9 (44%) patients under SAT had interrupted treatment at least once for 15 days or more during their treatment duration.

**Figure 1:**
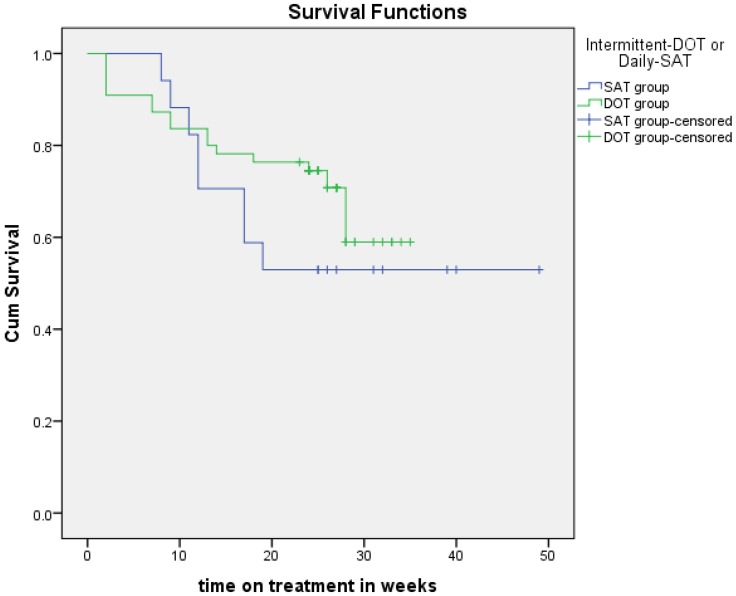
Probability of adverse outcomes in patients receiving Category-1 ATT under intermittent DOT or daily SAT.

Bivariate analysis showed that age, sex, TB site registration category and treatment outcome were not statistically different between the groups. The difference in treatment duration for patients receiving treatment was significant between the groups. Kaplan-Meier curves (Figure 1) showed no difference between the two groups.

## Discussion

The study indicates that complete tuberculosis treatment can be given in a low intensity conflict zone in India using a model of care alternative to DOT. A SAT model of care may be a feasible option to be adopted in similar settings, where DOT is difficult to be administered.

We observed that the duration of treatment for patients under DOT was shorter than under SAT with more frequent treatment interruptions among SAT patients. The small size of the study should be considered during interpretation of the analysis, however, this showed the operational reality of the programme. As per results of our study, we feel these were likely due to availability of fixed healthcare structure in the DOT area. When a patient under DOT missed a dose, they could visit the provider next day. However, patients under SAT received medication through a mobile clinic team, visiting the location every week. Hence, if a patient under SAT missed one appointment of mobile clinic, then they missed doses for the entire week, owing to more instances of interrupted treatment and longer duration of medication. Apart from duration of treatment, all of the patient and clinical characteristics were similar in patients under DOT or SAT, indicating comparable successful treatment outcome by either model of care.

Treatment outcomes in tuberculosis patients may depend on many factors like HIV-co infection, distance to health facility, literacy levels of the patients, marital status (in terms of treatment support), access to self-remedies/traditional healers, mode of administration of drugs etc. Some of them previously documented were intermittent vs daily dosages and comparison of fixed-dose-combination (FDC) with loose medications. Studies have reported similar sputum conversion rates in patients receiving intermittent or daily doses for tuberculosis treatment [11], with higher default and relapse among patients on intermittent treatment [12]. Single tablet FDC has been compared with loose pills for tuberculosis treatment [13], reporting similar treatment outcomes, however increased patient acceptability was associated with FDC [14]. Further, a study carried out by Nackers et al in Homa Bay, Kenya suggested self-administered therapy (SAT) with fixed dose combinations (FDC) helps in achieving appropriate adherence to treatment [15].

Our colleagues have reported experiences of an MSF tuberculosis treatment programme in Somalia where the need for DOT led the teams to create “TB villages” and “DOT corners” to ensure continuous provision of treatment [16]. We think that this was an interesting alternative to DOT. However, several different options should be explored to adapt to population needs in various contexts.

Appropriate monitoring of SAT for TB treatment, would require simple and validated tools [15] administered by trained staff. Counseling and treatment education should be provided to patients before initiation of treatment, to avoid relapse and loss to follow up during TB treatment on SAT [6]. In addition, efforts would be required for follow-up investigations, counseling and continuous drugs supply for the patients.

Apart from appropriate follow-up, security concerns and physical remoteness [17], [18] may account as major challenges for initiation and further completion of tuberculosis treatment. The SAT group in the study did not have any health structure nearby and thus had more instances of interrupted treatment, with longer duration of treatment in comparison to DOT group. Erratic, non-monitored use of medications in DOT or SAT models of care may lead to resistant forms of tuberculosis in the community.

The strength of this report was that we were able to study two different models of care for tuberculosis treatment, delivered by the same team side by side. This operational research project reflected the ground reality, as the teams were obliged to adopt different models-of-care depending on security and availability of health services. However, the study findings are limited by the small number of patients and therefore generalization or extrapolation of the results are not possible. There may be many other factors influencing tuberculosis outcome of these patients, however, it would be difficult to comment on these factors with routine programme data.

Tuberculosis resistance patterns at the population level are not available for most parts of India, including the study area. We were not able to study resistance patterns in our cohorts and we cannot comment on the patients on category II treatment (excluded from the analysis) nor to investigate treatment failures recorded in this programme. This is also one of the limitations of the study.

However, to our knowledge, this is the first study that reports on tuberculosis treatment outcomes among IDPs in a low-intensity chronic conflict zone in India.

## Conclusion

In conflict settings and extreme situations where there is no or limited access to functional healthcare care services, we have to find innovative ways of healthcare delivery. Self-administered-treatment strategies may serve as an alternative to DOTS in order to achieve universal coverage for tuberculosis treatment. The ‘one-size-fits-all’ strategy adopted by several national TB programmes may be revised with more flexible and context-adapted strategies and patient-centered approaches may be discussed and piloted.
